# Transcriptome profiling of immune responses to cardiomyopathy syndrome (CMS) in Atlantic salmon

**DOI:** 10.1186/1471-2164-12-459

**Published:** 2011-09-23

**Authors:** Gerrit Timmerhaus, Aleksei Krasnov, Pål Nilsen, Marta Alarcon, Sergey Afanasyev, Marit Rode, Harald Takle, Sven Martin Jørgensen

**Affiliations:** 1 Nofima AS, P. O. Box 5010, N-1432 Ås, Norway; 2 Department of Animal and Aquacultural Sciences, Norwegian University of Life Sciences, P. O. Box 5003, N-1432 Ås, Norway; 3 PHARMAQ AS, P. O. Box 267, N-0213 Oslo, Norway; 4 National Veterinary Institute, P. O. Box 750, N-0106 Oslo, Norway; 5 Sechenov Institute of Evolutionary Physiology and Biochemistry, Peterburg, Russia; 6 AVS Chile SA, Puerto Varas, Chile

## Abstract

**Background:**

Cardiomyopathy syndrome (CMS) is a disease associated with severe myocarditis primarily in adult farmed Atlantic salmon (*Salmo salar *L.), caused by a double-stranded RNA virus named piscine myocarditis virus (PMCV) with structural similarities to the *Totiviridae *family. Here we present the first characterisation of host immune responses to CMS assessed by microarray transcriptome profiling.

**Results:**

Unvaccinated farmed Atlantic salmon post-smolts were infected by intraperitoneal injection of PMCV and developed cardiac pathology consistent with CMS. From analysis of heart samples at several time points and different tissues at early and clinical stages by oligonucleotide microarrays (SIQ2.0 chip), six gene sets representing a broad range of immune responses were identified, showing significant temporal and spatial regulation. Histopathological examination of cardiac tissue showed myocardial lesions from 6 weeks post infection (wpi) that peaked at 8-9 wpi and was followed by a recovery. Viral RNA was detected in all organs from 4 wpi suggesting a broad tissue tropism. High correlation between viral load and cardiac histopathology score suggested that cytopathic effect of infection was a major determinant of the myocardial changes. Strong and systemic induction of antiviral and IFN-dependent genes from 2 wpi that levelled off during infection, was followed by a biphasic activation of pathways for B cells and MHC antigen presentation, both peaking at clinical pathology. This was preceded by a distinct cardiac activation of complement at 6 wpi, suggesting a complement-dependent activation of humoral Ab-responses. Peak of cardiac pathology and viral load coincided with cardiac-specific upregulation of T cell response genes and splenic induction of complement genes. Preceding the reduction in viral load and pathology, these responses were probably important for viral clearance and recovery.

**Conclusions:**

By comparative analysis of gene expression, histology and viral load, the temporal and spatial regulation of immune responses were characterised and novel immune genes identified, ultimately leading to a more complete understanding of host-virus responses and pathology and protection in Atlantic salmon during CMS.

## Background

Cardiomyopathy syndrome (CMS) is a severe cardiac disease affecting Atlantic salmon (*Salmo salar *L.). Since its first diagnosis in Norway 1985 [1], it has also been diagnosed in sea farms in Scotland, the Faroe island, Denmark and Canada [2]. CMS primarily affects farmed fish from 12 to 18 months after transfer to sea water [3,4], but cases of CMS in wild salmon have also been observed [5].

The diagnosis of CMS is based on cardiac histopathology, characterised by severe inflammation and necrosis of the spongy myocardium of the atrium and ventricle [6]. Inflammatory infiltrates consist of mononuclear cells, probably lymphocytes and macrophages. The compact layer of the ventricle is usually less affected, and always occurs later than changes in the spongious layer [6,7]. Farmed salmon suffering from CMS often lack clinical signs and may die suddenly due to rupture of the atrium or sinus venosus resulting in cardiac tamponade [1,6]. Other symptoms like skin haemorrhages, raised scales and oedema have also been reported [3,5]. At necropsy, ascitic fluid, fibrinous perihepatitis and blood clots on the liver and heart are typical findings [3,5,6]. The first study indicating a transmissible nature of the disease, showed typical cardiac lesions in salmon post-smolts six weeks post injection of cardiac and kidney homogenate from CMS-diseased fish [7].

Recently a novel virus associated with CMS was cultured and identified [8]. The proposed virus named piscine myocarditis virus (PMCV) is a double-stranded RNA virus with structural similarities suggesting assignment to the *Totiviridae *family. In this study, viral RNA could be detected by quantitative real-time RT-PCR (qPCR) from 2 weeks post challenge, peaking at 6-8 weeks post challenge, coinciding with the increase of histopathological lesions in the heart. Virus particles were also detected by *in situ *hybridization in degenerate and necrotic cardiac myocytes from field outbreaks of CMS.

In the present study, the same PMCV inoculum was used to experimentally reproduce CMS and to characterise the host immune response in infected salmon post-smolts. To gain an understanding of the immune response and host-virus interaction, a genome-wide approach based on oligonucleotide microarrays was used [9]. Six gene sets representing different arms of the immune response were identified, and temporal and spatial regulation was evaluated in combination with histology and relative quantification of viral RNA. The findings provide a comprehensive understanding of the immune response against PMCV in Atlantic salmon, and pathological and protective correlates thereof.

## Results

### Experimental CMS infection

**Figure 1:**
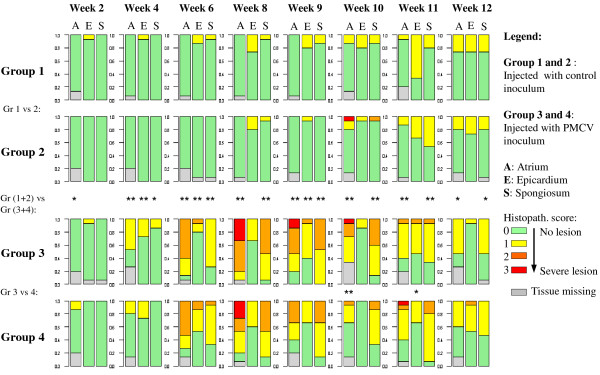
**Cardiac histopathology results of PMCV infected and control groups**. The proportions of histopathology scores in atrium (A), epicard (E) and spongious (S) were plotted as percentage bars. The bars represent the results of the 15 sampled fish per group and time point. The histopathology scores are shown as colours from green (score 0) to red (score 3), grey indicates missing tissue. Asterisks in the middle row represents the level of significance between groups (*/** = p < 0.05/0.01), according to two-sided *t-*tests. The row in the middle represents the difference between the two infected groups and the two control groups.

No mortality or clinical signs associated with CMS was observed. Potential contamination by other pathogens was excluded by qPCR for known viruses and bacteria from relevant organs and numbers of samples. Histopathological examination of heart was scored 0-3 according to severity of CMS lesions, as summarised in Figure 1. Results were used for evaluation of the infection challenge and for design of gene expression analyses. In control groups, one fish had moderate to severe cardiac lesions at 10 wpi, and was graded score 2 in the spongy layer of the ventricle and score 3 in the atrium. For all the other control fish, only score 0 and 1 were observed. No statistical difference between replicate control groups was found.

Groups receiving PMCV inoculum developed cardiac lesions consistent with CMS from 6 wpi and onwards. At 6 wpi, 63% of the infected fish had moderate lesions (score 2) in the atrium (percentages refer to observations, excluding missing values). Lesions were first found in the atrium and subsequently in the spongy layer of the ventricle. The peak of histopathological lesions was observed at 8 wpi, with moderate atrial lesions (score 2) in 36%, and severe lesions (score 3) in 32% of the fish. In the subsequent time points, fewer fish had cardiac lesions, and at 9, 10 and 11 wpi, respectively 7.4%, 4.3% and 3.8% of the fish were scored 3. At 12 wpi, only mild focal lesions (scores 0 and 1) were described in the atrium and spongy ventricle. In general, lesions were first found in the atrium and were more severe than in the spongious layer. Differences between group 3 and 4 were significant for atrial lesions at 10 wpi and epicardial lesions at 11 wpi. Lesions in atrium of control groups 1 and 2 versus infected groups 3 and 4 were statistically different for all time points except 12 wpi, with highest significance between 4 and 11 wpi (p < 0.01). A similar difference was found in spongious lesions with highest significance between 6 and 11 wpi. Lesions in epicardium differed significantly between infected and controls at 4, 6 and 9 wpi.

### Viral load

**Figure 2:**
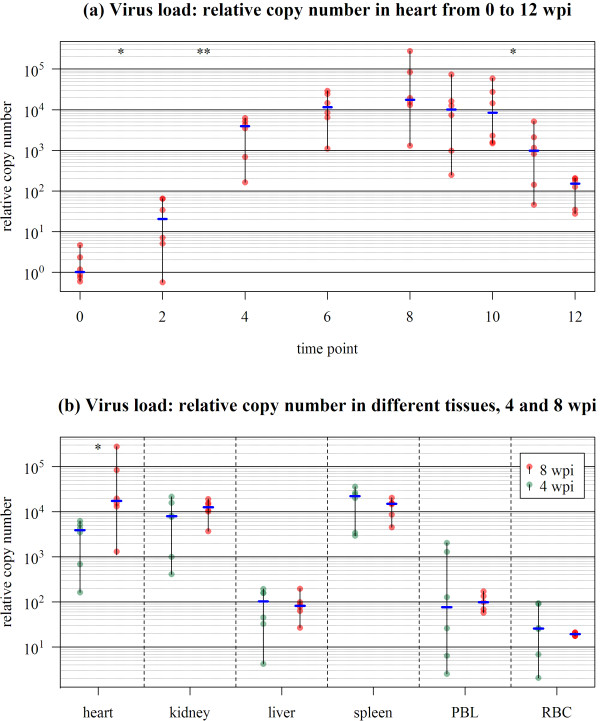
**Assessment of viral load by qPCR**. (a) Time course study in heart tissue: PMCV levels are shown as relative copy numbers for six individuals per time point. Copy numbers are relative to the median of the 0 wpi values which was set to 1. The black lines show the variance and blue bars represent the median. (b) PMCV levels in different tissues at 4 and 8 wpi. Relative copy numbers are shown as green dots (4 wpi) or red dots (8 wpi). Numbers are relative to median of 0 wpi values set to 1 (a). Levels of significant differences (*t*-test) were calculated for log-transformed values.

**Figure 3:**
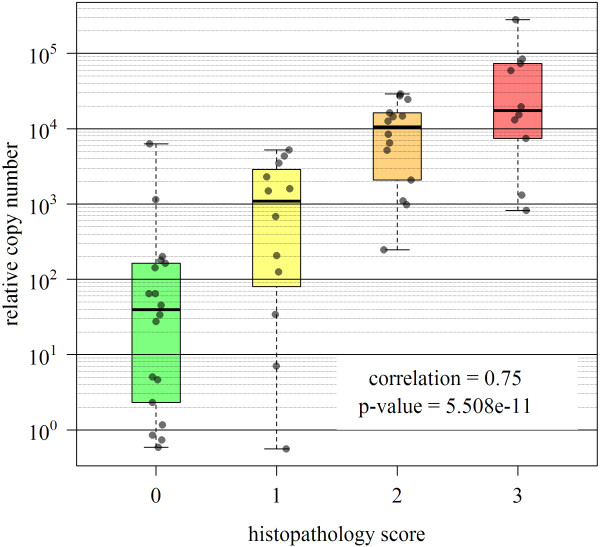
**Correlation between cardiac histopathology and viral loads**. Histopathology scores were correlated against relative copy numbers of PMCV in infected fish (n = 6) from all time points. Data points are plotted randomly across the box area in x-direction for better visualization. Correlation coefficient and p-value were calculated for log-transformed copy numbers.

PMCV levels were analysed by qPCR to document viral replication in heart during infection and in the different tissues at early infection (4 wpi) and peak pathology (8 wpi) stages (Figure 2). The same six individuals per time point as used for gene expression analyses were tested. Since 0 wpi and the two latest time points (11 and 12 wpi) were not included in microarray analysis, six randomly chosen samples from group 3 and 4 were tested respectively. At 2 wpi, 5 out of 6 fish were positive for viral RNA in heart (median of relative copy number = 20.5 fold, Figure 2a). Levels increased strongly until 4 wpi and then gradually until 6 wpi (median of relative copy number = 11, 583 fold), concurrent with the onset of histopathological changes. Levels reached a plateau phase between 6 and 10 wpi with no significant changes in viral RNA. From 10 to 11 wpi, levels were significantly reduced, indicating a clearance of virus. One week later (12 wpi), both viral load and individual variance were reduced. For most time points, individual variation in viral RNA was observed, analogous to the variation observed for histopathology score. Correlation between histopathology scores and viral C_T _levels in heart was highly significant (correlation coefficient: 0.75, p = 5.5 × 10^-11^) (Figure 3).

Comparison of viral loads between tissues showed highest and equal viral loads in heart, spleen and kidney (Figure 2b). Significantly lower and equal levels of viral RNA were found in blood cells (PBL and RBC) and liver. Except for heart (p = 0.030), viral loads were not significantly different between 4 and 8 wpi in any of the tissues investigated.

### Identification of gene sets representing immune pathways

**Figure 4:**
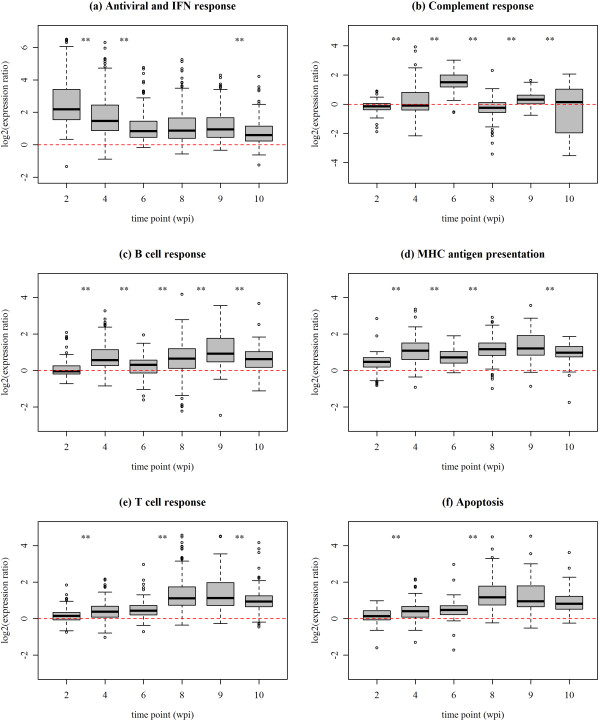
**Temporal regulation of immune responses in PMCV infected fish**. Box plots show log_2_-ER (expression ratios) for all genes included in the six gene sets (a-f) resulting from microarray analysis of infected versus control fish from the time course study in cardiac tissue. Boxes represent 50% of the values, while black bars mark the median log_2_-ER. Whiskers indicate the maximum length of 1.5 times the box length. Values beyond whiskers are plotted as circles. Significance levels of *t*-tests between time points are coded with asterisks: * = p-values between 0.01 and 0.05, ** = p-values < 0.01.

The main purpose of the gene expression study was to identify gene sets representing different immune pathways and characterise their regulation over the time course of CMS in the infected organs. Fish were challenged by injection to ensure simultaneous infection and virus dose. Since histomorphological changes were investigated in cardiac tissue, RNA from infected versus control heart samples from six time points (2, 4, 6, 8, 9 and 10 wpi) were used for microarray analysis. In order to examine responses in fish with similar disease status and infection level, individuals with highest histology scores and viral loads were selected from the time points when pathological changes were significant (6-10 wpi). After microarray experiments, 5712 differentially expressed genes with a mean log_2_-ER > |0.65| in at least one time point were selected. Genes implicated in different immune pathways were defined in the resulting list using the STARS software package [9], which contains custom annotation of genes on the microarray based on GO classes, KEGG pathways, mining of literature and public databases and experimental evidence (transcription profiles/meta-analysis). Further, immune genes were arranged in seven sets taking into account both functions and the expression profiles. Six gene sets (Additional file 1) showed differential expression between at least two subsequent time points (one-way ANOVA with Newman-Keuls test, Additional file 2), while one gene set (inflammatory components) was excluded since no significant temporal changes were found. The log_2_-ER for all genes per gene set and time point were combined from microarray results of the two sample pools (2, 6, 9, 10 wpi) and four sample pools (4 and 8 wpi). The resulting expression profiles of the six gene sets are shown as box plots in Figure 4. Gene composition and temporal regulation for each gene set is presented in the following section.

### Composition and temporal regulation of immune pathways

#### 1: Early antiviral and interferon response

**Figure 5:**
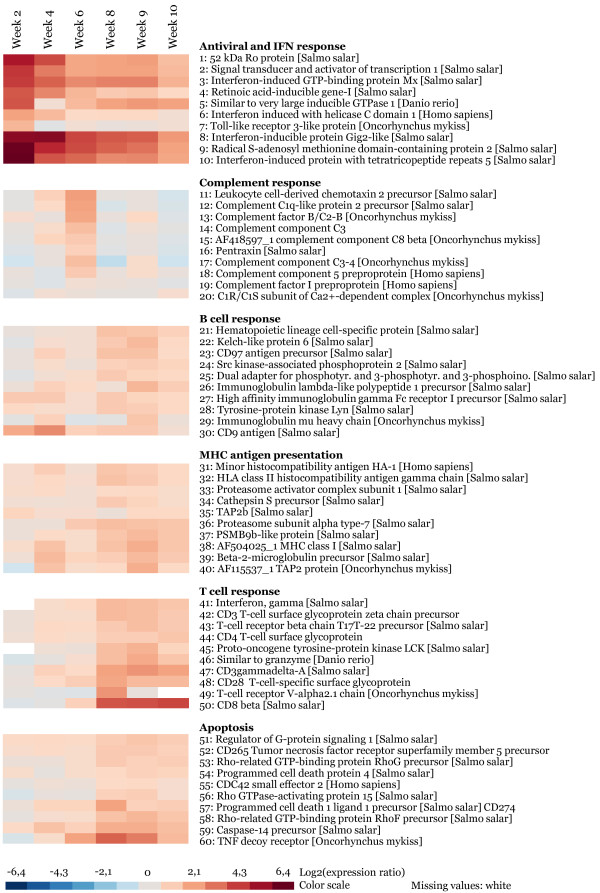
**Temporal regulation of representative genes**. Heat map showing the temporal expression of ten selected genes from each immune pathway (gene set) from Figure 4, as referred to in the Results section. Data are mean log_2_-ER of replicate pools of heart tissues from the six time points (2 to 10 wpi). Graded levels from gray to red indicate upregulation, and graded levels from gray to blue indicate downregulation. The scale of the colors is shown at the bottom of the figure.

This gene set included 85 genes associated with nonspecific innate immunity related to the early antiviral and interferon (IFN) responses. This also included predicted pattern recognition receptors (e.g. toll-like receptors and RIG helicases) and associated genes, and early induced virus-responsive genes known from other salmonid viral disease profiles in our microarray database (e.g. inflammasome/pyrin-like genes such as VHSV-induced and TRIM/RING finger genes). The expression profile showed strongest upregulation at the early stages which levelled off during infection (Figure 4a). A median log_2_-ER +2.1 at 2 wpi decreased to +0.7 at 6 wpi. This level remained unchanged until 9 wpi followed by a significant decrease to +0.5 at 10 wpi. A heat map showing the expression of ten genes is given in Figure 5. These were selected either by random or based on their functional importance as evidenced from other studies in fish or higher vertebrates. Early upregulation of the cytoplasmic RNA helicases *retinoic acid inducible gene I *(*rigI*) and *melanoma differentiation-associated gene 5 *(*mda5*) involved in sensing and degradation of viral RNA, as well as a gene similar to the membrane-bound *toll-like receptor 3*, implied activation of virus recognition receptors and antiviral signalling. Several genes known to be activated in response to IFN signalling were upregulated, such as *signal transducer and activator of transcription 1a *(*stat1a*), *myxovirus resistance gene Mx*, *interferon-inducible protein Gig2-like *and *radical s-adenosyl methionine domain-containing protein 2 *(*rsad2*) also known as *viperin*. A similar expression profile was also observed for a suit of genes known to be induced by IFN but with unknown roles in fish immunity, such as *interferon-induced protein with tetratricopeptide repeats 5 *(*ifit5*) and *very large inducible GTPase 1 *(*vlig1*). A transcript encoding the 52 kDa Ro protein was one of several TRIM/RING finger genes highly induced at 2 and 4 wpi, supporting the role of this multi-gene family in early virus recognition and host defence [10].

#### 2: Complement response

Twenty-two genes associated with the complement system were not differentially regulated at 2 wpi followed by a gradual upregulation from 4 wpi which peaked at 6 wpi (median log_2_-ER +1.5), concurrent with the onset of cardiac pathology. In subsequent time points expression levelled off, with a weak but significant induction at 9 wpi coinciding with pathology peak (Figure 4b). The upregulation at 6 wpi was significantly stronger compared to earlier and later time points. The heat map of representative genes (Figure 5) shows activation of genes with different roles in the complement system: Antigen: antibody-complex binding by C1q; activating enzymes C2b and C1r/s; membrane-binding proteins and peptide-inflammatory mediators C3a/4a/5a, C3, C3-4 and C5 pre-protein; and membrane-attack protein by C8b.

#### 3: B cell response

This gene set included 37 genes involved in differentiation and regulation of B cells and antigen recognition by immunoglobulins. The expression profile was characterised by upregulation at two time points; during early infection 4 wpi and at peak pathology 9 wpi (Figure 4c). The later peak was stronger with median log_2_-ER +0.93 compared to +0.57 at 4 wpi. The two peaks were separated by the complement activation at 6 wpi. Immunoglobulin-related genes, represented with 21 distinct transcripts, comprised a large part of this group (Additional file 1). Genes related to antigen receptor signalling included *hematopoietic lineage cell-specific (Lyn substrate 1) protein (hs1) *and *kelch-like protein 6 (klhl6) *(Figure 5). Similar function was predicted for several genes with Src homology-3/2 (Sh3/2) domains and activities, such as *src kinase-associated phosphoprotein 2 *(*skap2*) and *dual adapter for phosphotyrosine and 3-phosphotyrosine and 3-phosphoinositide *(*dapp1*). The *tyrosine-protein kinase lyn *also plays a regulatory role in B cell receptor response after antigen binding. The *CD9 antigen*, which is expressed in many B cell subsets and in plasma cells in mammals [11,12], was strongest induced at the early peak. The opposite was found for *CD97 antigen precursor*, suggesting that it may have a role in activated B and T cells.

#### 4: MHC antigen presentation

This gene set included 34 genes involved in processing and presentation of viral antigens via MHC class I and II. The expression profile was similar to that of B cell response, but with less difference in average induction levels between the two peaks at 4 and 9 wpi, respectively log_2_-ER +1.09 and +1.21 (Figure 4d). Besides, these genes were significantly upregulated already at the earliest time point (2 wpi). The gene set was dominated by genes related to the MHC class I pathway, such as antigen processing by proteasome components PSMBs/TAPs, and antigen presentation by the *MHC class I heavy chain *and light chain *beta-2-microglobulin *(Figure 5). Examples of MHC class II related genes were a salmon homologue to the *HLA class II histocompatibility antigen gamma chain *and *cathepsin s precursor*, a lysosomal cysteine peptidase involved in degradation of peptides for antigenic presentation on MHC class II molecules [13].

#### 5: T cell response

The fifth gene set included 69 genes with known or presumed roles in the regulation and effector functions of T lymphocytes. The expression profile showed a slight but significant upregulation from 2 to 4 wpi which increased by additional +1 median log_2_-ER at 8 wpi and reached maximum of +1.4 log_2_-ER induction at 9 wpi (Figure 4e). This peak coincided with highest levels of the MHC antigen presentation and B cell response genes, and the time points when viral load and cardiac pathology were peaking. From 9 to 10 wpi gene expression dropped significantly. All classes of effector T cells seemed to be activated from 8 wpi onwards; cytotoxic (CTL) cells by induction of *interferon gamma, granzyme *and *CD8 beta *and T helper cells by induction of *CD4 T cell surface glycoprotein *(Figure 5). Upregulation of other genes with common regulatory roles in T cell activation included CD3 antigens, T cell receptor genes, *CD28 T-cell specific surface glycoprotein *and the *proto-oncogene tyrosine-protein kinase lck*.

#### 6: Apoptosis

A group of 25 genes functionally linked to apoptotic pathways showed a coregulated expression pattern with the T cell response gene set, and was assumed to be involved in controlling cell death of T lymphocytes and/or host target cells, as their maximum induction coincided with the histopathology peak (Figure 4f). This gene set included several genes from the family of TNF receptors and caspases, with central roles in the execution phase of apoptosis (Figure 5). Interestingly, the majority of genes was linked to the family of Rho GTPases, with recently established roles in controlling T cell regulation and apoptosis, e.g. *rho-related GTP-binding protein RhoF *and *G precursors*, *CDC42 small effector 2*, *rho GTPase-activating protein 15*, *regulator of G-protein signalling 1*, and several genes related to the Ras superfamily (Additional file 1). Other important regulators of programmed cell death in immunity which were activated included the *tnf decoy receptor (tnfrsf6b) *and the *programmed cell death 1 ligand 1 precursor CD274*.

### Tissue regulation of immune pathways

**Figure 6:**
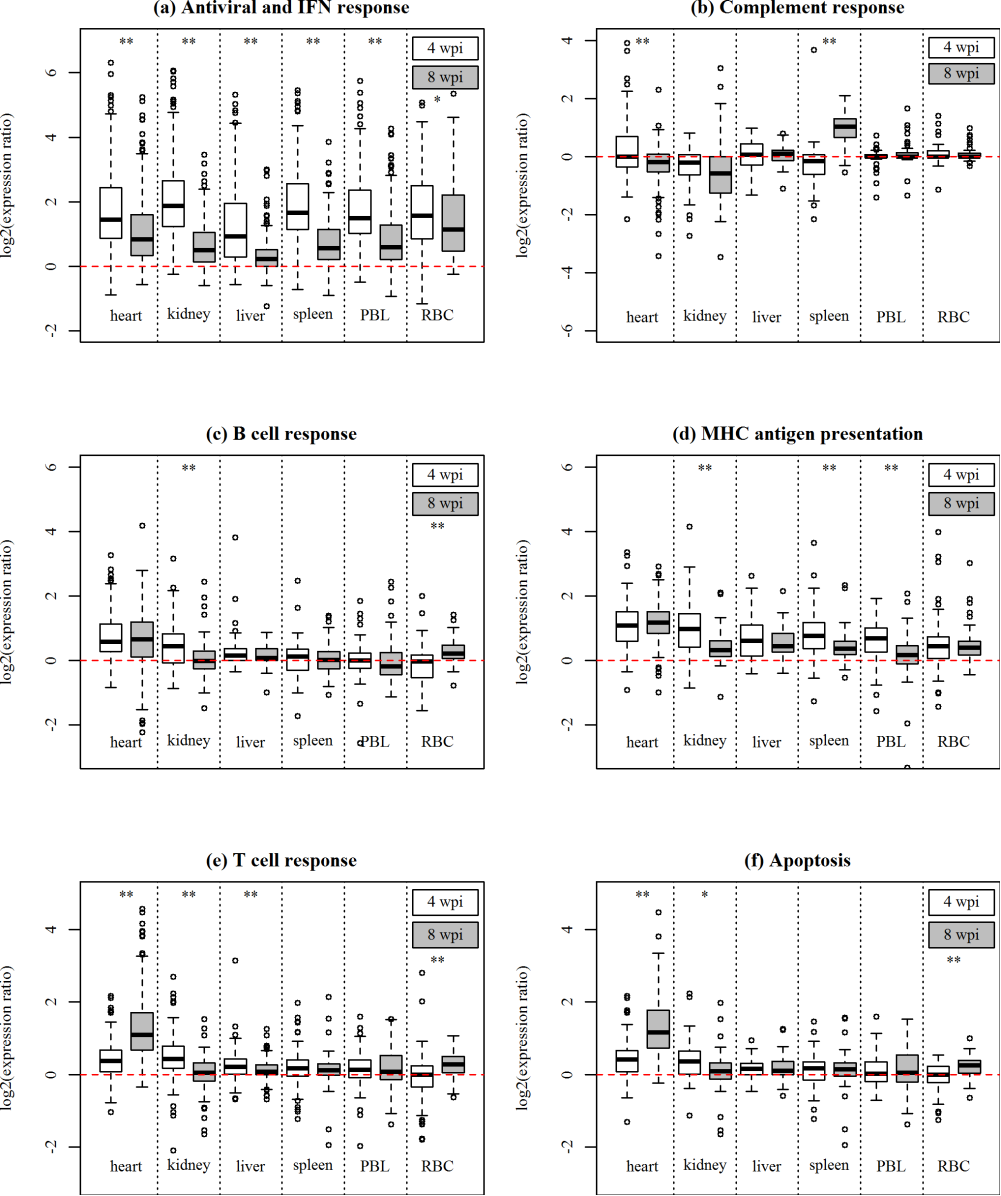
**Regulation of immune responses in different tissues**. Box plots show log_2_-ER (expression ratios) for all genes included in the six gene sets (a-f) resulting from microarray analysis of infected versus control fish from the study in different tissues and from two time points; early infection stage (4 wpi) and peak cardiac pathology/viral load (8 wpi). Details on the box plots are described in the legend of Figure 4.

Next, we analysed the tissue-specific features of immune transcriptome responses during CMS. Two RNA sample pools (n = 3 each pool, same individuals as analysed in the time course study) from the same organs as tested for viral load were analysed by microarrays from two time points; before the onset of cardiac pathology at 4 wpi and at peak of cardiac pathology/viral load at 8 wpi. The six gene sets outlined in the time course study were examined (Additional file 1), and their expression profiles are shown as box plots in Figure 6. Early antiviral and IFN-dependent genes were induced in all tissues, with significantly higher median log_2_-ER at 4 wpi compared to 8 wpi (Figure 6a). Levels at 4 wpi were similar in kidney, heart, spleen and blood, being lower in the liver. MHC antigen presentation also responded to infection in all examined tissues and, except for heart, levels were generally stronger at 4 versus 8 wpi (Figure 6d). The remaining functional groups showed restricted expression changes. The complement response was upregulated in spleen at the peak of pathology 8 wpi (Figure 6b). Genes associated with B cells were upregulated in heart and at both time points (Figure 6c). They also showed a weak but significant induction in kidney at 4 wpi and in RBC at 8 wpi. The T cell and apoptosis gene sets showed similar expression profiles, with induction in heart which was strongest at peak pathology 8 wpi when compared to 4 wpi (Figure 6e-f). In addition, a significant though relatively weak increase was found in RBC between 4 and 8 wpi. Similar to the B cell response, kidney showed a transient induction at 4 wpi.

### Real-time qPCR analyses

**Figure 7:**
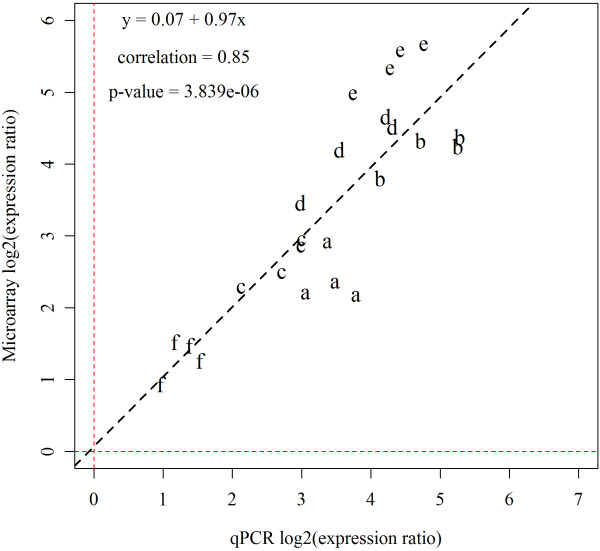
**Confirmation of microarray results by qPCR**. Expression levels of six genes were compared, as listed in Additional file 4. Log_2_-ER from microarrays was plotted on the y-axis, while log_2_-transformed fold changes from real-time qPCR were plotted on the x-axis. The four replicate pools of heart tissue from 4 wpi against control pools were measured for each gene. The dashed black line represents the regression function of the measured values. The function is shown in the top left corner of the plot. In addition, the Pearson correlation and the corresponding p-value are shown.

**Figure 8:**
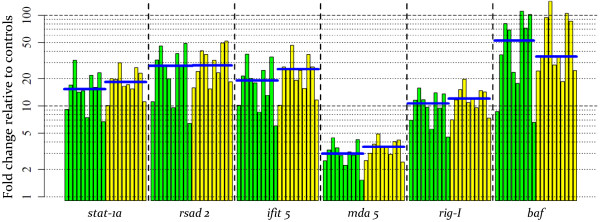
**Assessment of individual expression of antiviral genes**. Gene expression in 20 infected fish (from groups 3 + 4 at 4 wpi) is shown as fold change relative to uninfected controls based on real-time qPCR. The y-axis is plotted logarithmic. Bars are colored according to the histopathology score of atrium for respective fish; green = score 0, yellow = score 1. The median fold change for each histopathology score per gene is indicated with a blue line.

To verify the microarray results, six differentially expressed genes were analysed by qPCR in the four sample pools from 4 wpi. The results produced with two independent methods were in close concordance (Figure 7). The coefficient of linear regression was close to unity (0.97) while correlation and linear dependency were highly significant (Pearson r: 0.85, p = 3.8 × 10^-6^). The qPCR analyses also assessed the individual variation and relationship between viral load and gene expression changes at 4 wpi. Six genes encoding putative antiviral and IFN-dependent genes from gene set 1 were selected due to high induction levels at this early time point. Relative expression of *rig-I, mda5, stat1a, ifit5, rsad2 *and *baf *was determined in 20 individuals from CMS infected groups 3 and 4 versus the same control pool as used for the microarray experiments (n = 10) (Figure 8). These genes were strongly induced in all fish with median fold changes from +3 (*mda5*) to +52.5 (*baf*). At this time point, no significant histopathological changes were observed, and equal numbers of individuals had histopathology scores of 0 or 1. As expected, none of the analysed genes showed significantly different expression between fish with histopathological scores 0 and 1 (both corresponded to a normal state of heart). Viral load in heart varied between C_T _19-25 in these individuals, and gene expression levels and virus C_T _values were strongly correlated for all six genes (Table 1). This implied that genes were activated as a result of increased viral replication and suggested that they might represent markers of early infection status.

**Table 1:** Correlation between expression levels of antiviral genes and viral load.

Gene	Correlation coefficient	p-value
*stat-1a*	0.863419	0.001287

*rsad 2*	0.887746	0.000605

*ifit 5*	0.854039	0.001659

*rig-I*	0.909621	0.000261

*baf*	0.903799	0.000333

*mda 5*	0.836384	0.002561

mRNA levels (fold change relative to controls, real-time qPCR) of six genes (from early antiviral and IFN gene set) from 6 individual fish were tested for correlation (Pearson) against viral loads (non-normalized C_T _values, real-time qPCR) at 4 wpi.

## Discussion

This study addressed the temporal and spatial development of immune responses assessed by transcriptome changes during experimental piscine myocarditis virus infection. The regulation of immune pathways was compared to the disease status evaluated by histopathology and viral load, aiming at a comprehensive understanding of the host-virus interactions. These results provide a framework for in-depth functional studies on immunity and for evaluation of preventive strategies such as vaccination and nutritional intervention during CMS in Atlantic salmon.

### Challenge trial and infection

Since the discovery of CMS, its diagnosis has been based on clinical findings and cardiac histopathology [4]. A virus with structural similarities to the *Totiviridae *family named PMCV was recently identified as the presumed causative agent of CMS [8]. Thus, pathogenesis and disease progression can now be more thoroughly evaluated by combining virus-specific qPCR with histology. It should be noted that due to difficulties with PMCV cultivation *in vitro*, virus titration has not been successful yet (M. Rode, personal communication). Consequently, the relative expression of viral RNA in this study could not be related to actual numbers of viral particles. Cardiac histopathology showed moderate to severe lesions consistent with CMS (score 2 or 3) exclusively in infected groups, with only one exception in control groups at 10 wpi. Furthermore, replicate groups were very similar to each other with respect to histopathology score. Significant differences between group replicates were only observed between infected groups at two time points (atrium at 10 wpi and epicardium at 11 wpi). The differences between infected and control groups were mainly associated with lesions in the atrium and spongy layer of the ventricle, which were highly significant from 4 to 11 wpi and 6 to 11 wpi, respectively. As expected, no lesions were observed in the compact layer of the ventricle, and lesions in epicardium were less prominent. These results are coherent with the pathology as described from clinical outbreaks and previous challenge trials with CMS [4,7,8].

After the peak in histology score at 8-9 wpi, lesions declined gradually suggesting the onset of a recovery phase. This was supported from qPCR analysis of viral load which followed the same pattern: increased replication until 6 wpi followed by a plateau phase until 10 wpi, and finally decreasing levels to the end point of the challenge trial. Thus, the strong correlation between histopathology and viral load which peaked concurrently with the activation of T cell pathway genes, suggest that the observed cardiac lesions resulted from virus cytopathic effects and necrosis of infected myocytes, triggering an inflammatory response followed by activation of T-cell mediated immunity. Examination of viral loads across different tissues showed equally high levels of viral RNA in kidney and spleen as compared to heart, while liver and blood cells had lower levels. However, increased replication from 4 to 8 wpi was only observed in heart, supporting that this organ was the main site of virus propagation [8]. However, heart may not be the primary replication site, since viral RNA was detected in all tissues and blood cells early after infection. High infection levels in kidney and spleen are typical for viral diseases in **s**almon, and are probably related to their roles in attracting primary infected/antigen presenting cells and priming lymphocytes for specific immunity. From a challenge trial with the recently described piscine reovirus (PRV) [14], higher viral loads were found in spleen and kidney as compared to lower but equal levels between heart and liver [15]. While belonging to different families, both PRV and PMCV cause necrosis and inflammation in heart muscle. The lower levels of PRV in heart may reflect a more persistently infecting nature compared to PMCV [8,15].

While clinical CMS outbreaks typically give 5-20% mortality [4], no fish died in the present challenge trial. This suggested that during natural CMS outbreaks, either larger fish (or different life stages), higher numbers of infectious viral particles or possibly additional stressors must be present to give mortality. Coherence between distinct stress factors and viral infection resulting in higher mortality has been shown for other diseases in farmed Atlantic salmon [16,17]. An interesting observation was the high proportion of fish with no or moderate cardiac lesions at time points when viral loads and histopathological scores were significant. Thus, fish obviously exhibited different outcomes of infection. Comparison of these groups is currently under investigation. In the present study, fish with strongest pathology and viral infection were selected at each time point, in order to characterise immune responses at the transcriptome level in fish at similar stages of the disease process and with representative CMS pathology. Fish was challenged by injection to ensure simultaneous infection of all fish and since cohabitation had shown to give slower development of disease and weaker overall pathology ([8] and unpublished results). The individual qPCR analysis of six antiviral genes in 20 fish 4 wpi showed similar levels of upregulation, supporting that all fish had mounted equal antiviral responses following infection and were in similar disease state. This was further supported by a significant increase in viral loads of fish between week 2 and 4.

### Temporal development of immune responses

Early antiviral and IFN responses were activated at every time point and across tissues during infection. However, the overall expression profile showed declined mRNA levels with time in spite of increased virus replication. This contrasted the strong correlation between expression levels of six selected genes and viral load observed at 4 wpi, implying that activation of these genes with increased production of viral RNA was predominant at the early stage and possibly related to autocrine effects, such as pathogen recognition and induction of signalling pathways. The subsequent reduction in transcriptional activity might be due to increased paracrine effects of proteins in the induced innate responses, including both effector functions for clearance of virus and recruitment of immune cells for development of humoral and adaptive immunity. The IFN type I responses to different viral diseases have been reported in salmonids, with particular focus on IFN alpha and Mx protein [18-21]. We identified a suite of putative IFN-dependent genes with stronger upregulation which have been unknown or scarcely investigated in salmon. Most of these genes have shown responses to other viral diseases in salmon [9]. Strongest induction at 2 wpi was found for *ifit5 *and *rsad2*, also known as *viperin*. Both genes are known to be induced by IFN and involved in defence against viruses [22,23]. Little is known about *gig1*- and *gig2-like *genes in fish, but they were induced by viral infection in grass carp cells [24]. Members of this gene family were also strongly induced in rainbow trout 24 h post infection with the parasite causing whirling disease [25]. Four different genes from the tripartite motif (TRIM) family C-IV were also significantly induced over several time points. One of them, TRIM25, has been implicated in the RIG-I pathway by regulating the capability of RIG-I to activate type I IFN [26,27]. Several genes belonging to families of IFN-inducible GTPases were also early induced, including two transcript similar to very large inducible GTPase 1 (VLIG1) and eight transcripts similar to GTPase IMAP family member 7. The role of these novel GTPases in vertebrate infection is gaining interest [28,29].

Proteins of the complement system bind and opsonize viral particles, marking them for phagocytosis by APCs. Binding to antigen-antibody complexes makes the complement system a bridge between the innate and the adaptive immune system. This is in line with results of the present study, where upregulation of complement genes at 6 wpi took place shortly after the first activation of B cell- and MHC antigen presentation genes and the onset of cardiac histopathology. In subsequent time points, activation of the adaptive immune response was most prominent. This distinct sequence of immune events was evidence for a coordinated regulation of responses, and the 'bridging' role of the complement system between the early innate response and the fully activated adaptive response. Coincidence with the first occurrence of moderate cardiac lesions (histopathology score 2), suggests complement genes as candidates disease markers for the early clinical stage of CMS.

The immediate activation of antigen presentation as has been observed during early virus infection in salmon [19], was supported by the upregulation of proteasome and MHC class I pathway genes that coincided with the early IFN/antiviral response at 2 wpi. This was analogous to the typical development of an adaptive immune response: while IFNs are strongest activated and elicit antiviral effects very early after infection, they also have an activating effect on antigen processing and presentation [30]. Activation of antigen presentation is also the first step in the cellular immune response mediated by B and T lymphocytes. The first peak of B cell activity was detected at 4 wpi following the typical pattern of a humoral immune response in teleost fish, usually expected between 4 and 6 weeks after infection [31]. However, the co-regulated B cell- and MHC antigen presentation genes showed a biphasic expression, with a second and even stronger activation at 8 and 9 wpi when the clinical signs were also peaking. This observation is probably explained by the higher influx of leukocytes and level of inflammatory reactions in heart tissue as supported by histology. Interestingly, stronger second peak of induction occurred after the activation of complement genes at 6 wpi. This may indicate that a potential humoral response based on antibody-dependent cellular cytotoxicity and virus neutralization is complement-dependent. Future development of tools for assessment of virus-specific antibody titers may confirm this. Most of the representative genes (Figure 5) followed the typical regulatory pattern for B cell and antigen presentation components. For example, the strongest induction of CD9 was found at 2 and 4 wpi, before the strongest T cell activation was detected. It has been shown that CD9 is induced downstream of the antigen receptor during T-independent humoral B cell response [32]. However, the majority of genes showed highest upregulation in heart at peak pathology and viral load, 8 and 9 weeks after infection, indicating their role in B cell responses and presentation of viral antigens to effector T cells. One example was CD97, a surface protein of both B and T lymphocytes which is expressed at low levels in inactive cells but rapidly induced after activation [33]. Thus, it can be used as a marker of general activation of lymphocytes. In this study it was a representative marker gene for the overall expression profile of B/T lymphocyte-related responses.

The co-regulatory pattern of T cell- and apoptosis-related genes correlating with histopathology score was a prominent feature of the immune response in CMS hearts. Control of cell death by apoptosis is a fundamental process for regulation of the T cell response and for maintaining homeostasis in the immune system after it has expanded to combat infections [34]. Importantly, dysfunctional control of T cell function and apoptosis is associated with immunopathology [35]. Thus, the apoptosis-related profile coinciding with the T cell profile in this study may represent novel genes involved in regulation of effector function and controlled cell death of T cells in salmon. Of particular interest were several genes encoding TNF-related proteins and programmed cell death ligand 1 (aka B7-H1/CD274). The dominance of genes encoding Rho GTPases was interesting, since they have been implicated in the regulation of TCR signaling, T cell cytoskeletal reorganization, T cell migration and T cell apoptosis [36]. It seemed to be a borderline from 6 to 8 wpi when expression of T cell/apoptosis pathways was significantly induced, coinciding with the first occurrence of histopathology scores 3 and virus C_T _values below 20. According to this pattern, the first severe inflammation and cytopathic effects caused by the virus (histopathology score 2) at 6 wpi was probably the priming event for a strong influx of lymphocytes to the infected heart tissue. Cardiac elevation of mRNA levels for CD8, granzyme and IFN gamma at 8-10 wpi indicated activity of cytotoxic CD8^+ ^T cells. Genes encoding CD4 were also induced, but at lower levels. One week after elevation of T cell activity (9 wpi, median of relative fold change of PMCV = 10, 021) viral load and histopathology score were decreasing (10 wpi, median PMCV fold change = 8, 404), and the first significant decrease was evident at 11 wpi (median PMCV fold change = 983). This indicated that the cellular effector response mediated by T cells, and in particular CD8^+ ^T cells, was contributing to a successful clearance of the virus infection. Among other interesting genes in this group, TNF decoy receptor showed the highest correlation versus histopathology score and viral load. However, the function of this receptor in salmon immunity is not known.

### Tissue regulation of immune responses

The systemic induction of early antiviral and IFN-dependent genes was expected, given the observed replication of PMCV in all tissues and the fact that most of these genes are presumably activated in the presence of viral RNA. The stronger induction at early infection 4 wpi compared to peak viral load 8 wpi was common for all tissues and blood, and has already been discussed. The functional relation between IFNs and MHC antigen presentation pathways was supported by their similar expression profiles across tissues and time points. The only exception was the cardiac expression of the latter, which was equally induced between time points (4 and 8 wpi). Proteasome and MHC components are commonly induced by IFNs during viral infection [19,37]. In addition to heart tissue, where pathology developed, these genes were equally induced in spleen and kidney at 4 wpi supporting the importance of these tissues for lymphocyte maturation and priming of the immune response [38]. The observation that this induction was not in sync with viral load may further suggest that these responses were time-dependent, e.g. related to the stage of disease rather than viral load and pathology. Little is known about the expression of complement components in Atlantic salmon during viral infections. In common carp and channel catfish, the highest expression of complement was found in the liver [39,40]. In humans, liver is also the main source of complement component C3, but production in macrophages and endothelial cells has also been shown [41]. During CMS, complement genes were only activated in extrahepatic tissue and more specifically in cardiac tissue, where virus infection was most prominent. Interestingly, complement genes were induced in the spleen during clinical phase, suggesting that splenocytes (e.g. macrophages) represent an important source of complement and can play a role in this response in salmonid virus infection. This induction of complement was also independent of viral load, which was equal between 4 and 8 wpi. In humans, the complement component C3 has an important role in regulating the maturation of B cells in the spleen [42]. Thus, the induction of splenic complement might reflect signalling events between activation of antigen presenting cells such as B cells and possibly production of virus-specific antibodies. However, more research is needed to understand this process. Tissue regulation of adaptive immune responses as represented by expression of B cell, T cell and apoptosis gene sets shared some common features. Most notable was the opposite regulation of these responses in heart and kidney between the early and clinical stage, which was characterised by an induced expression from 4 to 8 wpi in heart, and reduced expression from 4 to 8 wpi in kidney. Interestingly, twelve genes (among them CD8, CD37, granzyme and TNF decoy receptor) showed no regulation in heart but induced expression in kidney at 4 wpi. On the contrary, at 8 wpi induction was restricted to heart while no expression changes were found in kidney. This could be evidence for an early clonal expansion and maturation of effector T cells in kidney which then migrated to the heart for elimination of virus-infected cells four weeks later. The adaptive immune responses in kidney was activated at the early stage of infection despite equally high levels of viral RNA at both 4 and 8 wpi, further suggesting a specific role for kidney in the early priming and maturation of cellular immunity.

## Conclusions

We used oligonucleotide microarrays to assess transcriptome changes in Atlantic salmon experimentally infected with PMCV, inducing cardiac pathology consistent with CMS and transient viraemia. From comparative analysis of gene expression, histology and viral load, the temporal and spatial regulation of immune responses were characterised and novel immune genes identified, ultimately leading to a more complete understanding of host-pathogen responses and pathology and protection in Atlantic salmon during CMS.

## Methods

### Experimental infection and sampling

The infection trial was performed at VESO Vikan (Veterinary Science Opportunities, Namsos, Norway), a GLP-certified research station for infectious challenge experiments on aquatic organisms. The trial was approved by The National Animal Research Authority http://www.fdu.no according to the 'European Convention for the Protection of Vertebrate Animals used for Experimental and other Scientific Purposes' (EST 123). The experimental design with selection of sampling times and PMCV inoculum was based on results from two previous pilot trials [7] (and unpublished results). From both of these experiments, histopathological lesions associated with CMS were significant from week 6 until week 10 post challenge (injection). Therefore, in the present study we sampled weekly from 8 until 12 weeks post challenge, aiming to cover the period with CMS pathology. Biweekly sampling after infection (2, 4, 6 wpi) was done in order to cover the early phase before pathology. Unvaccinated Atlantic salmon (*Salmo salar *L., standard strain from Aqua Gen AS) were smoltified (seawater-adapted) according to standard procedures and kept at 12°C under standardised conditions (light, feeding, water flow, salinity and fish density). Fish were acclimatised in respective tanks for at least one week before challenge.

The trial was conducted in four separated tanks; one infected and one control group in duplicates. Each tank contained 120 fish with an average weight of 50 g at the beginning of the experiment. Injection of PMCV was performed after sedation (benzocaine, 30-40 mg L^-1^). Infected groups received an intraperitoneal (i.p.) injection dose (0.2 ml) of a supernatant from a GF-1 cell line (derived from the fin tissue of orange-spotted grouper, *Epinephelus coioides *[43]) infected with PMCV as described [8]. In short, heart tissue from freshly dead Atlantic salmon was collected from a clinical field outbreak of CMS (diagnosed by histopathological examination, score > 3). Tissue was homogenised, centrifuged to remove cellular debris (4000 g at 4°C for 20 min) and filtrated (0.22 μm filter) before inoculation onto GF-1 cells. Cells were grown in plug seal cap culture vessels at 15°C in L-15 supplemented with 1% L-glutamine (2 mM), 0.1% gentamicin sulphate (50 μg ml^-1^) (all from Sigma Aldrich, St. Louis, USA) and 10% fetal bovine serum (Invitrogen, CA, USA). Cytopathic effect (CPE) was evident by accumulation of cytoplasmic vacuoles from 6 until 21 days post inoculation, when supernatant and cell lysate were harvested. CPE was reproduced when passaged onto fresh cells. Inoculation of cells with heart tissue homogenate prepared from healthy Atlantic salmon (confirmed by histopathology, score 0) did not give CPE. Tanks with control groups were injected i.p. with the same dose of conditioned medium from uninfected cell culture prepared as described above. Both PMCV and control inoculums were tested negative for salmonid alphavirus subtype 3, infectious pancreatic necrosis virus, infectious salmon anaemia virus, piscine reovirus and grouper nervous necrosis virus by qPCR.

An overview of samplings and analyses is given in Additional file 3. Tissues and blood were sampled at eight time points: 2, 4, 6, 8, 9, 10, 11 and 12 wpi, in order to cover early infection phase (biweekly sampling) and clinical phase with improved coverage (weekly sampling). In addition, samples were taken from fish before the experiment started (0 wpi). From each time point, 15 fish from each of the four tanks were sedated (as described above) and euthanized by decapitation. Standardised samples from heart, mid-kidney, liver and spleen were snap-frozen in liquid nitrogen for RNA, and fixed in formalin (10% neutral phosphate-buffered) for histology. Blood was sampled from the caudal vein in heparinized vacutainers and kept on ice. Peripheral blood leukocytes (PBL) were separated from red blood cells (RBC) as described [44] and stored at -80°C until RNA was extracted.

### Histopathology

Formalin-fixed heart samples were prepared by paraffin wax embedding and standard histological techniques [45]. Sections were stained with haematoxylin and eosin. From each fish, a longitudinal section of the whole heart was evaluated. All cardiac compartments were examined and classified histologically using a visual analogue scale. Atrium, epicardium, compact and spongy layers of the ventricle were graded from 0 to 3 according to the severity of the lesions [7]. Score 0 and 1 was considered normal, with no histopathological findings (score 0), or a single or few focal lesions (score 1). Score 2 represented several distinct lesions and increased mononuclear infiltration. Score 3 represented multifocal to confluent lesions in > 50% of tissue and moderate to severe leukocyte infiltration. Sections were coded and evaluation was randomised and blinded.

### RNA extraction

Tissue samples for microarray hybridization and qPCR were stored at -80°C prior to RNA extraction. Standardised tissue sections of 10 mg from each organ and 5-10 × 10^6 ^blood cells (PBL and RBC) were prepared under sterile/RNase-free conditions. Tissue sections from heart consisted of an equal mix of ventricle and atrium with all compartments included. Frozen sections were transferred directly to 1 ml chilled TRIzol (Invitrogen) in 2 ml tubes with screw caps (Precellys^®^24, Bertin Technologies, Orléans, France). Two steel beads (2 mm diameter) were added to each tube and tissue was homogenized in a Precellys^®^24 homogenizer for two times 25 sec at 5000 rounds per minute with a pause of 5 sec between rounds. Blood samples were homogenized in 1 ml chilled TRIzol by repetitive pipetting up and down. RNA was extracted from the homogenized tissues using PureLink RNA Mini kits according to the protocol for TRIzol-homogenised samples (Invitrogen). The concentration of extracted total RNA was measured with a NanoDrop 1000 Spectrometer (Thermo Scientific, Waltham, MA, USA). The integrity of total RNA was determined using an Agilent 2100 Bioanalyzer with RNA Nano kits (Agilent Technologies, CA, USA). Samples with RNA integrity number (RIN) of 8 or higher were accepted.

### Design of microarray experiments

An overview of microarray analyses is given in Additional file 3. The salmonid oligonucleotide microarray (SIQ2.0, NCBI GEO platform GPL10679) was used, consisting of 21 K features printed in duplicates on 4 × 44 K chips from Agilent Technologies [9]. Two-color design was used, where pooled infected fish labelled with fluorescent Cy5 dye and non-infected pooled control fish from the same time point labelled with Cy3 dye were competitively hybridised on the array. Microarray hybridizations were divided in two experimental lines. The first was a time course study in heart tissue from 2, 4, 6, 8, 9 and 10 wpi. These time points were selected based on the results from histopathological examination (Figure 1), and covered the early infection phase and peak of cardiac pathology. From each time point, biological replicates of test (infected) samples consisted of two RNA pools with each pool consisting of three individual fish. For time points 4 and 8 wpi, representing respectively the early and clinical phase of infection, two new pools were added, each consisting of two fish (different than those used in the first two pools). The individual fish were selected for maximum heart histopathology score at the time points when this was significant (from 6 wpi onwards, see Figure 1). Reference samples were pooled RNA (equimolar amounts of total RNA) from eight to ten fish from groups 1 and 2 (non-infected controls) per each time point. The second experimental line focused on tissue responses in mid-kidney, liver, spleen, PBL and RBC (in addition to heart as described above) at the early and clinical phase of infection, respectively 4 and 8 wpi. Similar to the time course study, biological replicates were two pools of RNA, each consisting of three individual fish, per each tissue and time point. The two pools were RNA from the same six individuals as used for the time course study. Reference sample pools were prepared in the same manner, with RNA from the same non-infected control individuals as used for the time course study. Recording of microarray experiment metadata was in compliance with the Minimum Information About a Microarray Experiment (MIAME) guidelines [46].

### Microarray hybridization and data processing

Unless specified otherwise, all reagents and equipment used for microarray analyses were from Agilent Technologies according to manufacturer's protocol. Labelling and amplification of RNA was performed on 500 ng total RNA using Quick Amp Labeling Kits, Two-Color and RNA Spike-In Kits, Two-Color. For fragmentation of labelled RNA, the Gene Expression Hybridization Kit was used. Hybridizations were performed for 17 hours in an Agilent hybridization oven set to 65°C with a rotation speed of 10 rounds per minute. Arrays were washed for one minute with Gene Expression Wash Buffer I at room temperature, and one minute with Gene Expression Wash Buffer II at 37°C. Slides were scanned immediately after washing using a GenePix Personal 4100 A scanner (Molecular Devices, Sunnyvale, CA, USA) at 5 μm resolution and with manually adjusted laser power to ensure an overall intensity ratio close to unity between Cy3 and Cy5 channels and with minimal saturation of features. The GenePix Pro software (version 6.1) was used for spot-grid alignment, feature extraction of fluorescence intensity values and assessment of spot quality. After filtration of low quality spots, data were exported into the STARS platform [9] for data transformation, normalization and quality filtering. The values in spot replicates were averaged, Lowess normalization of log_2_-expression ratios (ER) was performed, and differentially expressed genes (DEG) were selected based on mean log_2_-ER > |0.65| in at least one time point and tissue, spot signal quality threshold, number of positive spots and one-sample *t*-test (p < 0.05, H_0_: log_2_-ER = 0). Corrections for false discovery rate were not employed as previous microarray studies in Atlantic salmon have demonstrated them to be overly conservative [47,48]. The final list of DEG used for further analysis included 5712 genes. Data was submitted to GEO (submission number GSE28843).

### Gene sets and annotations

For this work, functional subgroups or gene sets were compiled from the list of 5712 differentially expressed genes (Additional file 1). These were created by the use of the STARS software package customized for mining of microarray gene expression data [9]. STARS contain custom annotations of genes on the microarray based on GO classes, KEGG pathways, mining of literature and public databases and experimental evidence (transcription profiles/meta-analyses).

### Quantitative real-time RT-PCR

The following section relates to the analysis of host gene expression. Experiments were conducted according to the MIQE guidelines [49]. Synthesis of cDNA was performed on 0.2 μg DNAse-treated total RNA (Turbo DNA-freeTM, Ambion, Austin, TX, USA) using the TaqMan^® ^Gold Reverse Transcription kit (Applied Biosystems, Foster City, CA, USA) in 25 μl reactions with random hexamer priming according to manufacturer's protocol. Complementary DNA was stored undiluted at -80°C in aliquots to avoid repeated freeze-thawing. To avoid risk for presence of residual DNA contamination, control reactions without RT was tested on respective tissues and qPCR primers were possibly designed to span introns. Oligonucleotide primers were designed with the program eprimer3 from the EMBOSS program package (version 5.0.0, http://emboss.sourceforge.net/). Amplicon size was set to 80-160 and melting temperature to 59-61°C. Primers were purchased from Invitrogen (Additional file 4). *In silico *analysis of gene targets was performed using a customised program for BLAST and sequence alignments [9]. PCR amplicon size and specificity were confirmed by gel electrophoresis and melting curve analysis (Tm calling; LightCycler 480, Roche Diagnostics, Mannheim, Germany). QPCR was conducted using 2× SYBR^® ^Green Master Mix (Roche Diagnostics) in an optimised 12 μl reaction volume, using 5 μl of 1:10 diluted cDNA, and primer concentrations of 0.42 μM. PCR reactions were prepared manually and run in duplicates in 96-well optical plates on a Light Cycler 480 (Roche Diagnostics) with the following conditions: 95°C for 5 min (pre-incubation), 95°C for 5 sec, 60°C for 15 sec, 72°C for 15 sec (amplification, 45 cycles) and continuous increase from 65°C to 97°C with standard ramp rate (melting curve). Cycle threshold (C_T_) values were calculated using the second derivative method. For evaluation of the results, the mean of duplicates was used. Duplicate measurements that differed more than 0.5 C_T _values were removed and reanalysed. Relative expression ratios of test samples versus the average of the controls were calculated according to [50]. Elongation factor 1α (GenBank ID: BT072490.1) was used as reference gene [51], and was found to be stably transcribed in control and test samples according to the BestKeeper software [52]. The efficiency of the PCR reactions was estimated for all primer pairs by six times 1:5 dilution series of a cDNA mix of all used samples. The efficiency values were estimated by using the LightCycler^® ^480 Software (version 1.5.0.39). All measured efficiencies were between 1.905 and 1.999.

### Viral load

Relative quantification of PMCV was employed by qPCR on RNA isolated as described above from selected samples (heart; weeks 0, 2, 4, 6, 8, 9, 10, 11, 12, n = 6, kidney/liver/spleen/PBL/RBC; weeks 4 and 8, n = 6). Each sample's RNA concentration was normalized to 62.5 ng per 20 μl cDNA synthesis reaction, which was part of the SuperScript^® ^III Platinum^® ^Two-Step qRT-PCR Kit with SYBR^® ^Green (Invitrogen). In order to reduce secondary structures, RNA was heat denatured at 95°C - 5 min and then cooled down to 4°C prior to addition of RT enzyme and master mix. cDNA synthesis reaction conditions and temperature cycling were kept in line with manufacturer's guidelines. qPCR was performed on each sample in triplicate reactions containing 12.5 μl 2× Platinum^® ^SYBR^® ^Green qPCR SuperMix-UDG, ROX reference dye was added to the master mix to give a final reaction concentration of 50 nM, 1.25 μl 6 μM ORF2-3F (5'-GGAAGCAGAAGTGGTGGAGCGT-3') and 1.25 μl 6 μM ORF2-3R (5'-CCGGTTTTGCGCCCTTCGTC-3'). Ten μl 1:10 dilution of cDNA was added per reaction. The reaction conditions were UDG-incubation at 50°C for 2 min, activation of the hot-start polymerase at 95°C for 2 min, followed by 45 cycles of 95°C for 15 sec, primer annealing for 15 sec and extension for 45 sec at 60°C. Melting curve analysis was performed to confirm formation of expected amplicon. The viral loads were expressed as a relative copy number with non-infected controls (0 wpi) set to 1, calculated by the formula 2^(C_T(0wpi median)_- C_T(sample)_).

### Statistical analyses

Histopathology scores and pair-wise comparison of gene sets were tested for significant differences by an independent two-sample t test using the function *t*-test() in the R software STATS-package (version 2.10.1, http://www.cran.r-project.org/). In addition, one-way ANOVA followed by Newman-Keuls test was used to assess differences between time points and tissues for each gene set. Correlations and respective p-values were calculated by the cor.test() function in R. For all tests, significance levels of the resulting p-values with p < 0.05 and p > 0.01 are marked with single asterisk (*), and p < 0.01 are marked with double asterisk (**) in all figures. The function of the regression line and the respective p-value for the confirmation of the microarray experiments by qPCR were calculated by the lm()-function ("linear model") in R.

## Competing interests

The authors declare that they have no competing interests.

## Authors' contributions

GT and SMJ drafted the manuscript. GT, SMJ and AK conducted gene expression analysis. MR, HT and SMJ designed the challenge test. MR and PN prepared PMCV inoculum and analysed viral load. MA performed histopathological analysis. SA developed the software for processing of microarray data and performed parts of the statistical analyses. All authors have read and approved the final manuscript.

## Supplementary Material

Additional file 1 **Complete list of immune gene sets**. Gene composition and respective log_2_-ER for the six gene sets representing immune pathways regulated over time (in heart) and in different tissues, as referred to in the Results section. Click here for file

Additional file 2 **Results from ANOVA on gene sets**. Results of one-way ANOVA with Newman-Keuls test for the log_2_-ER values of the six gene sets from time points and tissues. Click here for file

Additional file 3 **Experimental outline**. Overview of sampling strategy and number of biological replicates for the different analyses. Click here for file

Additional file 4 **Real-time qPCR primers used in the study**. The first column refers to the letters used in Figure 7 for plotting of expression values per each gene. Click here for file
