# Supplementary material for: Transcriptome profiling of immune responses to cardiomyopathy syndrome (CMS) in Atlantic salmon
Source: BMC Genomics. 2011 Sep 23;12:459. doi: 10.1186/1471-2164-12-459 (PMC3196748; doi:10.1186/1471-2164-12-459)
Supplement: Additional file 4 — Real-time qPCR primers used in the study. The first column refers to the letters used in Figure 7 for plotting of expression values per each gene. [file 1471-2164-12-459-S4.DOC]

| **Conf** | **Best BLAST hit** | **ID, GenBank ID** | **Amplicon length** | **Forward**  **5’ 🡪 3’** | **Reverse**  **5’ 🡪 3’** |
| --- | --- | --- | --- | --- | --- |
| a | Signal transducer and activator of transcription 1 isoform alpha [Homo sapiens] | Stat1a  GQ325309.1 | 127 | CGGTGGAGCCCTACACTAAG | GGGATCCTGGGGTAGAGGTA |
| b | Barrier-to-autointegration factor [Salmo salar] | Baf  BT059902.1 | 112 | ACAGACCCCTCATCATCCTG | CGGTGCTTTTGAGAAGTGGT |
| c | Dead/h (Asp-Glu-Ala-Asp/His) box polypeptide rig-i [Homo sapiens] | Rig-I  FN178459.2 | 142 | GACGGTCAGCAGGGTGTACT | CCCGTGTCCTAACGAACAGT |
| d | Interferon-induced protein with tetratricopeptide repeats 5 [Salmo salar] | Ifit5 BT046021.1 | 118 | CAGAGAGGTGCCAGGCTAAC | TGCACATTGACTCTCCTTGG |
| e | Radical s-adenosyl methionine domain-containing protein 2 [Salmo salar] | Rsad2  BT047610.1 | 113 | GTACCGCAGATGCACAACAC | TTGACACTGCTTGGAGTTGC |
| f | Interferon induced with helicase c domain 1 [Homo sapiens] | Mda5  FN396357.1 | 116 | CAGAGGTGGGGTTCAATGAT | AGCTCGCTCCACTTGTTGAT |
|  | Elongation factor 1 alpha [Salmo salar] | Ef1a  BT072490.1 | 77 | CACCACCGGCCATCTGATCTACAA | TCAGCAGCCTCCTTCTCGAACTTC |
